# Breakfast consumption is positively associated with nutrient adequacy in Canadian children and adolescents

**DOI:** 10.1017/S0007114514002190

**Published:** 2014-09-08

**Authors:** Susan I. Barr, Loretta DiFrancesco, Victor L. Fulgoni

**Affiliations:** 1 Department of Food, Nutrition and Health, University of British Columbia, 2205 East Mall, Vancouver, BC, CanadaV6T 1Z4; 2 Source! Nutrition, Toronto, ON, Canada; 3 Nutrition Impact LLC, Battle Creek, MI, USA

**Keywords:** Dietary surveys, Nutrition assessment, Breakfast, Population studies

## Abstract

Although breakfast is associated with more favourable nutrient intake profiles in children, limited data exist on the impact of breakfast on nutrient adequacy and the potential risk of excessive intakes. Accordingly, we assessed differences in nutrient intake and adequacy among breakfast non-consumers, consumers of breakfasts with ready-to-eat cereal (RTEC) and consumers of other types of breakfasts. We used cross-sectional data from 12 281 children and adolescents aged 4–18 years who took part in the nationally representative Canadian Community Health Survey, 2004. Mean nutrient intakes (obtained using a multiple-pass 24 h recall method) were compared among the breakfast groups using covariate-adjusted regression analysis. Usual nutrient intake distributions, generated using the National Cancer Institute method, were used to determine the prevalence of nutrient inadequacy or the potential risk of excessive intakes from food sources alone and from the combination of food plus supplements. Of these Canadian children, 10 % were breakfast non-consumers, 33 % were consumers of RTEC breakfasts and 57 % were consumers of other types of breakfasts. Non-consumption of breakfast increased with age (4–8 years: 2 %; 9–13 years: 9 %; 14–18 years: 18 %). Breakfast consumers had higher covariate-adjusted intakes of energy, many nutrients and fibre, and lower fat intakes. The prevalence of nutrient inadequacy for vitamin D, Ca, Fe and Mg (from food alone or from the combination of food plus supplements) was highest in breakfast non-consumers, intermediate in consumers of other types of breakfasts and lowest in consumers of RTEC breakfast. For vitamin A, P and Zn, breakfast non-consumers had a higher prevalence of nutrient inadequacy than both breakfast groups. The potential risk of excessive nutrient intakes was low in all groups. Efforts to encourage and maintain breakfast consumption in children and adolescents are warranted.

The importance of breakfast in contributing to the nutrient intakes of children and adolescents has been recognised for decades, and has been the topic of numerous reviews^(^ ^1^ ^–^ ^4^ ^)^. These reviews and studies published more recently^(^ ^5^ ^–^ ^7^ ^)^ have indicated that breakfast consumption is frequently associated with higher energy and nutrient intakes, and that the overall nutrient profile appears most favourable among those who consume breakfasts that include ready-to-eat cereal (RTEC). Although most studies have been conducted in the USA, research conducted with children from other countries has yielded similar findings^(^ ^6^ ^,^ ^8^ ^–^ ^11^ ^)^. Despite these positive associations of breakfast consumption with nutrient intake, 20 % of 9- to 13-year-old children and 32 % of 14- to 18-year-old adolescents studied in the 1999–2006 National Health and Nutrition Examination Survey did not eat breakfast^(^ ^5^ ^)^, and the frequency of breakfast skipping has increased between the 1960s and the 1990s^(^ ^12^ ^)^.

It is probable that in many cases, higher nutrient intakes associated with breakfast and RTEC consumption translate to improved nutrient adequacy (where adequacy is defined as meeting nutrient requirements). Nevertheless, it is conceivable that higher nutrient intakes might have no impact on adequacy (if intakes of almost everyone met or exceeded the requirements) and could even increase the potential risk of adverse effects from excessive intakes. However, to date, most studies have not reported the association between breakfast consumption, or the type of breakfast consumed, and dietary nutrient adequacy as measured using the dietary reference intake (DRI) framework for dietary assessment^(^ ^13^ ^)^. Using this framework, the prevalence of dietary inadequacy can be estimated for most nutrients as the proportion of a population's usual nutrient intake distribution that falls below the estimated average requirement (EAR). If intakes are assessed accurately, one would expect that proportion of the population below the EAR to approximate the proportion of the population who do not meet their requirement for the physiological criterion of adequacy used to set the EAR^(^ ^13^ ^)^. Similarly, the prevalence of the potential risk of adverse effects from excessive nutrient intakes can be estimated as the proportion of the usual nutrient intake distribution that exceeds the tolerable upper intake level (UL). Because vitamin and mineral supplements may contribute to dietary adequacy as well as the potential risk of dietary excess^(^ ^14^ ^–^ ^16^ ^)^, it is important to account for supplement intake when assessing these parameters. Yet almost all studies examining the contribution of breakfast consumption have reported intakes from food sources alone. Accordingly, the purpose of the present study was to assess the differences in nutrient intake and adequacy among Canadian children and adolescents classified as breakfast non-consumers, consumers of breakfasts with RTEC and consumers of other types of breakfasts, considering intakes from food sources alone and from the combination of food sources and supplements. We hypothesised that breakfast consumption would be associated with a lower prevalence of nutrient inadequacy as assessed using the DRI framework, and would have little or no impact on the prevalence of the potential risk of excessive nutrient intakes.

## Methods

### Data source and participants

The present study used data from the Canadian Community Health Survey 2.2 (CCHS 2.2), a cross-sectional, nationally representative survey conducted by Statistics Canada in 2004^(^ ^17^ ^,^ ^18^ ^)^. The survey included a 24 h dietary recall, which was followed by a general health questionnaire assessing sociodemographic characteristics, physical activity, smoking, supplement use and food security, among other variables. Interviews were done by a proxy (parent or legal guardian) for children under the age of 6 years, with a parent or guardian (joint responses) for children aged 6 to 11 years, and by the respondent alone for individuals aged 12 years and above. In all cases, a parent or legal guardian consented to the participation of the child. Henceforth, ‘respondent’ or ‘participant’ is taken to include ‘proxy’ where appropriate. The target population for the survey was all individuals living in private dwellings in the ten Canadian provinces, which represented about 98 % of the Canadian population. The sampling strategy was designed to be representative in terms of age, sex, geography and socio-economic status. The response rate for the survey was 76·5 %, and a non-response adjustment was applied to the survey weights. Ethical approval for population health surveys conducted by Statistics Canada is based on the authority of the Statistics Act of Canada^(^ ^17^ ^,^ ^19^ ^)^. Approval to conduct the analyses reported herein was received from the Statistics Canada Research Data Centre Program^(^ ^20^ ^)^.

The present study included data from respondents aged 4 to 18 years who were not pregnant or lactating (*n* 12 281). Breakfast consumption was self-reported and included any foods or beverages consumed at an eating occasion that the participant defined as breakfast during the 24 h dietary recall^(^ ^18^ ^)^. Those who did not report any items as breakfast were classified as breakfast non-consumers. Those who consumed RTEC as a component of breakfast were classified as RTEC-breakfast consumers, and those whose breakfasts did not include RTEC were classified as other-breakfast consumers.

### Nutrient intakes

Dietary intake data were obtained using a slightly modified version of the US Department of Agriculture Automated Multiple-Pass Method for 24 h dietary recalls^(^ ^18^ ^,^ ^21^ ^)^. All respondents completed a 24 h dietary recall in person with a trained interviewer, and a subset of approximately 30 % completed a second 24 h dietary recall by telephone 3 to 10 d later. Nutrient contributions from foods were based on data from the Canadian Nutrient File, version 2001b^(^ ^22^ ^)^, which were primarily derived from the US Department of Agriculture Nutrient Database for Standard Reference 13^(^ ^23^ ^)^. The Canadian Nutrient File also includes Canadian-specific values for foods, including RTEC, which have differing composition in Canada than in the USA. In Canada, the nutrients that may be added to breakfast cereals (e.g. thiamin, niacin, vitamin B_6_, pantothenic acid, folate, Mg, Fe and Zn) and the amounts to be added are specified by the Food and Drugs Regulations^(^ ^24^ ^)^. For most nutrients, the amount that may be added is modest ( < 10 % of the daily value per serving), although it is somewhat higher for some nutrients (e.g. thiamin and Fe). In contrast, in the USA, voluntary fortification of breakfast cereals falls under food safety regulations and a fortification policy statement^(^ ^25^ ^)^. Generally, a wider array of nutrients and higher amounts (up to 100 % of the daily value per serving) can be found in US breakfast cereal products.

The general health component of the CCHS 2.2 included questions on supplement use^(^ ^18^ ^)^. Respondents who had taken a vitamin or mineral supplement within the past month were classified as supplement users. These individuals were asked to locate their supplement containers (from which the interviewer recorded complete product information) and provided details on the frequency of supplement use during the past month. For each nutrient, the average daily amount consumed as a supplement was included as a variable in the CCHS 2.2 data file.

### Estimation of usual nutrient intake distributions

Estimates of usual intake for each nutrient were determined using the National Cancer Institute method for a single dietary component^(^ ^26^ ^)^, using data from the second 24 h dietary recall to adjust for within-individual variation. Based on intake on the day of the first 24 h dietary recall, separate usual intake distributions were generated for each nutrient by DRI age/sex group for breakfast non-consumers, RTEC-breakfast consumers and other-breakfast consumers. As has been described elsewhere^(^ ^27^ ^)^, balanced repeated replication was used to create variance estimates, standard errors of percentiles and probabilities of meeting a DRI.

The prevalence of nutrient inadequacy was estimated as the proportion of respondents with usual intakes below the age/sex-specific EAR for thiamin, riboflavin, niacin (as niacin equivalents), folate (as dietary folate equivalents (DFE, where 1 DFE = 1 μg food folate or 0·6 μg folic acid from fortified foods))^(^ ^28^ ^)^, Ca, P, Mg, Zn, vitamin A (as retinol activity equivalents) and vitamins B_6_, B_12_, C and D^(^ ^13^ ^)^. Vitamin E was not included in the CCHS 2.2 data file, as the 2001b version of the Canadian Nutrient File was substantially incomplete for this nutrient. The probability method was used for Fe, which does not have a symmetrical requirement distribution^(^ ^29^ ^)^; accordingly, we used the requirement distributions published by the Institute of Medicine^(^ ^29^ ^)^. The prevalence of the potential risk of excessive nutrient intakes was estimated as the proportion of respondents with usual intakes above the age/sex-specific UL for folic acid, Ca, P, Fe, Zn, and vitamins B_6_, C and D^(^ ^13^ ^)^. Vitamin A, niacin and Mg were not examined for this analysis. The UL for vitamin A and niacin are based on preformed retinol and nicotinic acid or nicotinamide, respectively, and these nutrient forms are not available in the CCHS 2.2 data file. The UL for Mg is based on Mg added as a fortificant to foods and used as a supplement, but since Mg added to foods is also not available in the CCHS 2.2 data file, we could not assess intakes above the UL. Both nutrient inadequacy and the potential risk of excessive nutrient intakes were assessed for intakes from food sources alone and for total intakes from food sources plus supplements. The total intake distributions were generated for each breakfast group and age/sex group by adding each individual's daily supplemental intake of the selected nutrient (averaged over the past month) to their usual nutrient intake based on food sources alone^(^ ^30^ ^)^.

### Statistical analyses

Statistical Analysis Software (version 9.3; SAS Institute, Inc.) and SUDAAN (version 10.0; RTI International) were used to analyse the data. All analyses were adjusted for the complex CCHS 2.2 sampling design using appropriate sample weights and the MISSUNIT option in SUDAAN due to a large number of cases where only one stratum within a primary sampling unit was encountered during balanced repeated replication. This option then calculates variance contribution using the difference from the overall mean of the population. Means, percentages and standard errors were obtained using the PROC DESCRIPT procedure. Covariate-adjusted mean nutrient intakes among the three breakfast groups were compared using regression analysis (i.e. using the PROC REGRESS procedure), with *P*< 0·05 (Bonferroni-adjusted *P*< 0·0167) to assess the significance of multiple comparisons. Covariates included in the analysis were energy intake, age, sex, race, food security, language spoken at home and supplement use. Physical activity and sedentary activity (‘screen time’) could not be included as covariates because they were not assessed in children under 6 years of age, and different assessment questions were used in those aged 6 to 11 years *v.* those 12 years and above. Moreover, the age groups for activity assessment did not correspond to the DRI age groups. Similarly, smoking was not included as a covariate because it was queried only in those aged 12 years and above. To address the potential impact of omitting these covariates, separate analyses of nutrient intakes by breakfast group were conducted with and without their inclusion in children aged 6 to 11 and 12 to 17 years. We also examined whether observed differences in daily nutrient intakes between the RTEC-breakfast and other-breakfast groups were primarily due to the differences in intakes at breakfast, or were associated with the differences in dietary intakes during the rest of the day. To do this, nutrient intakes at breakfast and during the rest of the day (excluding breakfast) were compared between these two groups. Finally, the prevalence of nutrient inadequacy and the potential risk of excessive intakes were compared among the three breakfast groups using a *Z*-test and *P*< 0·05 (Bonferroni-adjusted *P*< 0·0167) to assess significance.

## Results

### Participant characteristics

Nationally weighted demographic characteristics are shown in Table 1 for the group as a whole and for each of the three breakfast groups. Breakfast non-consumers were the eldest of the three groups, and were less likely to use supplements than both groups of breakfast consumers. Among those aged 12 years and above, breakfast non-consumers had higher proportions classified as physically inactive and a higher average daily ‘screen time’ than breakfast consumers. They were also the most likely to smoke. Those consuming RTEC breakfasts were the youngest of the three groups and were more likely to be male than those consuming other types of breakfasts. Among those aged 12 years and above, RTEC-breakfast consumers had the lowest proportion who smoked and the highest proportion classified as physically active. Physical activity was also higher among RTEC-breakfast consumers aged 6 to 11 years compared with the other two groups. Finally, a smaller proportion of those consuming other types of breakfasts spoke English at home, compared with the other two groups. The proportions of consumers who were white, food secure and lived in an urban setting did not differ by breakfast group.

**Table 1:** Demographic characteristics of Canadian children and adolescents aged 4–18 years by breakfast group* (Mean values, standard errors and percentages)

Characteristics	All (*n* 12 281)	No breakfast (*n* 1471)	Other breakfast (*n* 6920)	RTEC breakfast (*n* 3890)
	%	sem	%	sem	%	sem	%	sem
Age (years)								
Mean	11·2	14·0^a^	11·2^b^	10·4^c^
sem	0·1	0·2	0·1	0·1
Male (%)	51·5	0·7	52·0^a,b^	2·3	48·7^a^	1·0	56·2^b^	1·3
Physical activity (h/week), age 6–11 years†								
Mean	12·8	11·0^a^	12·6^a^	13·4^b^
sem	0·2	0·6	0·2	0·2
Physical activity category (%), age 12–18 years†								
Active	41·0	1·0	35·7^a^	2·4	40·0^a^	1·4	46·0^b^	1·9
Moderate	25·6	0·9	23·8	2·1	26·6	1·2	24·4	1·7
Inactive	33·4	1·0	40·5^a^	2·4	33·4^b^	1·4	29·6^b^	1·7
‘Screen time’ (h/d), age 6–11 years‡								
Mean	2·6	2·7	2·6	2·5
sem	0·05	0·2	0·1	0·1
‘Screen time’ (h/d), age 12–17 years‡								
Mean	2·7	3·0^a^	2·6^b^	2·8^b^
sem	0·04	0·1	0·05	0·1
Dietary supplement use (% yes)	34·2	0·7	26·0^a^	2·0	34·2^b^	1·0	36·6^b^	1·3
Food Secure (% yes)§	91·2	0·4	90·0	1·2	92·0	0·6	90·2	0·8
Urban (%)	81·3	0·7	82·5	1·7	81·0	0·8	81·3	1·2
Smoking (% smokers), age 12–18 years	6·8	0·5	14·9^a^	2·0	6·4^b^	0·7	2·9^c^	0·4
White (%)	80·0	0·7	80·1	1·9	80·1	0·9	79·9	1·2
English spoken at home (%)	70·0	0·7	73·5^a^	2·4	66·5^b^	1·0	75·2^a^	1·2

RTEC, ready-to-eat cereal included with breakfast.

^a,b,c^Mean values within a row with unlike superscript letters were significantly different (*P*< 0·05; Bonferroni-adjusted *P*< 0·0167).

* Data were obtained from the Canadian Community Health Survey, Cycle 2.2.

† Different questions were used to assess physical activity in children aged 6–11 *v.* 12–18 years. Physical activity was not assessed in children under the age of 6 years.

‡ Different questions were used to assess ‘screen time’ (time spent on computers, playing video games and watching television) in children aged 6–11 *v.* 12–17 years. Screen time was not assessed in those aged < 6 or ≥ 18 years.

§ For respondents aged ≤ 17 years, a knowledgeable adult member of the household was asked about the food security questions. Those aged 18 years responded on their own behalf.

**Fig. 1:**
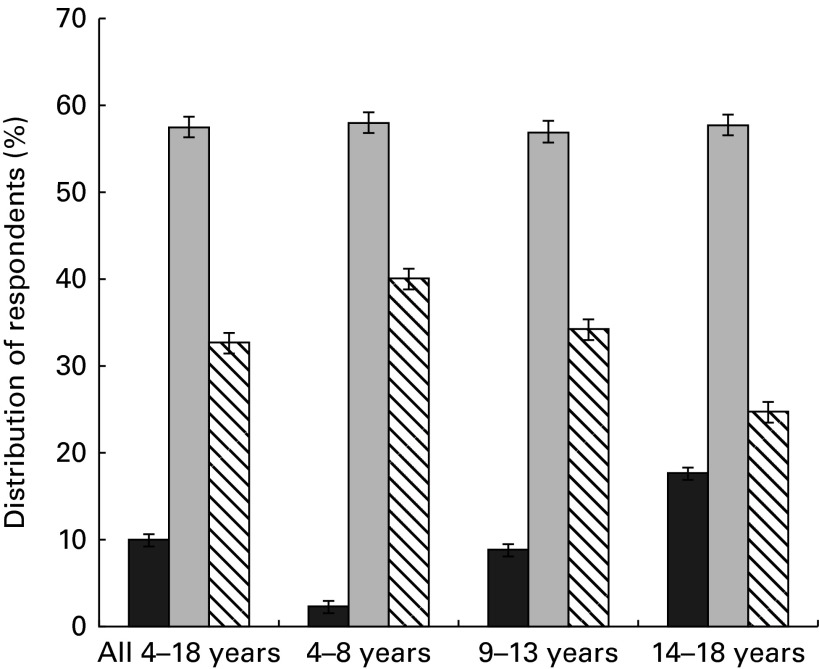
Distribution of respondents by breakfast group and age. 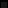, No breakfast; 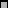, other types of breakfast; 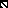, breakfast that included ready-to-eat cereal. Data were obtained from the Canadian Community Health Survey Cycle 2.2.

Overall, 10 % of Canadian children did not eat breakfast, 33 % consumed a breakfast that included RTEC and 57 % consumed other types of breakfasts (Fig. 1). The proportion of those who skipped breakfast increased progressively across the age groups from 2 % (4–8 years) to 9 % (9–13 years) to 18 % (14–18 years) (*P*< 0·001), whereas the corresponding proportions of those consuming RTEC breakfasts decreased (*P*< 0·001). There were no changes with age in the proportion of those who consumed other types of breakfasts.

### Nutrient intakes

The mean daily nutrient energy intake was lower in breakfast non-consumers than in both groups of breakfast consumers (Table 2). With regard to nutrients, several patterns of differences in energy-adjusted intakes were observed for the group as a whole: (1) stepwise significant increases in the intakes of carbohydrate, fibre, thiamin, vitamin B_6_, vitamin D, Ca, Fe, Mg, P and K among breakfast non-consumers, other-breakfast consumers and RTEC-breakfast consumers; (2) stepwise significant decreases in the intakes of total fat, saturated fat and monounsaturated fat across the three groups; (3) significantly higher intakes of vitamin A, folate and vitamin C in both groups of breakfast consumers compared with non-consumers; (4) significantly lower intakes of polyunsaturated fat and cholesterol, and significantly higher intakes of sugars and riboflavin in RTEC-breakfast consumers compared with the other two groups; and (5) a significantly higher intake of niacin in other-breakfast consumers compared with breakfast non-consumers, neither of which differed significantly from RTEC-breakfast consumers. Intakes of protein, vitamin B_12_, Zn and Na did not differ among the breakfast groups. In general, similar patterns of differences were observed among males and females when examined separately (Table 2), as well as among those aged 4–8, 9–13 and 14–18 years (data not shown), although the differences were not always significant.

**Table 2:** Nutrient intakes of Canadian children and adolescents aged 4–18 years by breakfast group* (Weighted means with their standard errors)

	All breakfast group	Male breakfast group	Female breakfast group
	None (*n* 1471)	Other (*n* 6917)	RTEC (*n* 3890)	None (*n* 731)	Other (*n* 3335)	RTEC (*n* 2191)	None (*n* 740)	Other (*n* 3582)	RTEC (*n* 1699)
Nutrients	Mean	sem	Mean	sem	Mean	sem	Mean	sem	Mean	sem	Mean	sem	Mean	sem	Mean	sem	Mean	sem
Energy (kJ)	7736^a^	188	9380^b^	75	9619^b^	105	8221^a^	266	10 401^b^	29	2535^b^	121	7209^a^	222	8355^b^	84	8468^b^	134
Protein (g)	80·0	1·3	81·2	0·5	80·8	0·8	92·0	2·0	90·7	0·8	91·2	1·3	67·1^a^	1·6	71·2^b^	0·6	69·4^a,b^	0·8
Fat (g)	84·3^a^	1·1	80·3^b^	0·4	73·4^c^	0·5	91·6^a^	1·4	89·0^a^	0·6	82·2^b^	0·8	76·5^a^	1·6	71·2^b^	0·5	64·1^c^	0·7
SFA (g)	29·1^a^	0·4	27·8^b^	0·2	26·3^c^	0·3	31·8^a^	0·6	30·8^a^	0·3	29·4^b^	0·4	26·2^a^	0·7	24·6^a^	0·3	22·9^b^	0·4
MUFA (g)	33·6^a^	0·6	31·5^b^	0·2	28·0^c^	0·2	36·4^a^	0·7	35·1^a^	0·3	31·5^b^	0·4	30·5^a^	1·0	27·7^b^	0·2	24·2^c^	0·3
PUFA (g)	13·6^a^	0·3	13·2^a^	0·1	11·8^b^	0·2	14·5^a^	0·4	14·5^a^	0·2	13·1^b^	0·2	12·5^a^	0·6	11·8^a^	0·2	10·5^b^	0·2
Cholesterol (mg)	257^a^	9	256^a^	3	207^b^	3	305^a^	14	292^a^	5	238^b^	5	207^a^	10	218^a^	4	173^b^	4
Carbohydrate (g)	288^a^	3	299^b^	1	317^c^	1	316^a^	4	327^a^	2	344^b^	2	260^a^	4	270^b^	1	288^c^	2
Sugars (g)	138^a^	2	135^a^	1	145^b^	2	150^a,b^	4	149^a^	2	158^b^	2	125^a,b^	3	120^a^	1	131^b^	2
Fibre (g)	13·2^a^	0·3	14·9^b^	0·1	16·1^c^	0·2	14·0^a^	0·4	15·7^b^	0·2	17·4^c^	0·3	12·3^a^	0·4	14·0^b^	0·2	14·8^c^	0·3
Vitamin A (μg RAE)	560^a^	19	647^b^	9	644^b^	11	581^a^	30	692^b^	14	699^b^	15	537	26	599	11	584	15
Thiamin (mg)	1·5^a^	0·02	1·7^b^	0·01	2·2^c^	0·03	1·6^a^	0·04	1·8^b^	0·02	2·5^c^	0·04	1·4^a^	0·03	1·5^b^	0·01	1·9^c^	0·04
Riboflavin (mg)	2·0^a^	0·06	2·1^a^	0·01	2·3^b^	0·03	2·3^a^	0·08	2·3^a^	0·02	2·6^b^	0·05	1·8^a,b^	0·10	1·9^a^	0·02	2·0^b^	0·04
Niacin (mg NE)	34·5^a^	0·6	36·0^b^	0·2	36·0^a,b^	0·4	39·2	0·9	40·0	0·4	40·2	0·6	29·5^a^	0·7	31·9^b^	0·3	31·3^b^	0·4
Vitamin B_6_ (mg)	1·5^a^	0·03	1·6^b^	0·01	1·8^c^	0·02	1·7^a^	0·05	1·8^a^	0·02	2·0^b^	0·03	1·3^a^	0·04	1·5^b^	0·02	1·6^c^	0·03
Folate (DFE)	404^a^	8	462^b^	4	458^b^	5	435^a^	12	498^b^	6	492^b^	7	373^a^	10	424^b^	5	424^b^	7
Vitamin B_12_ (μg)	4·1	0·2	4·0	0·1	4·1	0·1	5·0	0·4	4·6	0·1	4·7	0·2	3·0^a^	0·1	3·5^b^	0·1	3·4^a,b^	0·1
Vitamin C (mg)	129^a^	6	156^b^	2	148^b^	3	129^a^	7	162^b^	4	156^b^	4	130	9	148	3	140	4
Vitamin D (μg)	5·3^a^	0·2	6·0^b^	0·1	6·9^c^	0·1	6·1^a^	0·2	6·8^b^	0·1	7·6^c^	0·2	4·5^a^	0·3	5·2^b^	0·1	6·1^c^	0·2
Ca (mg)	974^a^	24	1042^b^	9	1192^c^	15	1075^a^	35	1148^a^	15	1310^b^	19	872^a^	31	928^a^	12	1063^b^	24
Fe (mg)	12·4^a^	0·2	13·1^b^	0·1	18·2^c^	0·2	13·8^a^	0·3	14·3^a^	0·1	20·0^b^	0·2	10·8^a^	0·2	11·8^b^	0·1	16·1^c^	0·3
Mg (mg)	257^a^	3	285^b^	2	306^c^	2	283^a^	5	308^a^	2	335^c^	3	230^a^	4	260^b^	2	275^c^	3
P (mg)	1301^a^	18	1371^b^	8	1463^c^	12	1472^a^	26	1519^a^	12	1626^b^	16	1121^a^	24	1215^b^	10	1285^c^	17
Zn (mg)	10·8	0·2	10·9	0·1	11·0	0·1	12·4	0·3	12·1	0·2	12·4	0·2	9·0^a^	0·2	9·6^b^	0·2	9·4^a,b^	0·1
Na (mg)	3187	59	3204	24	3125	31	3486	80	3513	35	3463	45	2864	87	2877	31	2761	37
K (mg)	2647^a^	43	2889^b^	18	2991^c^	26	2915^a^	60	3123^b^	27	3290^c^	37	2367^a^	61	2638^b^	24	2664^b^	32

RTEC, ready-to-eat cereal included with breakfast; RAE, retinol activity equivalents; NE, niacin equivalents; DFE, dietary folate equivalents.

^a,b,c^Mean values within a row with unlike superscript letters were significantly different (*P*< 0·05; Bonferroni-adjusted *P*< 0·0167).

* Data were obtained from the Canadian Community Health Survey, Cycle 2.2, adjusted for energy (for nutrients), age, sex, race, dietary supplement use, food security and language spoken at home.

Analyses similar to those reported in Table 2 were also conducted separately for children aged 6 to 11 and 12 to 17 years to examine the potential impact of adding covariates that could not be included in the primary analysis: physical activity; ‘screen time’; and smoking (12–17 years only). Of the 156 comparisons (two age groups, the twenty-six nutrients included in Table 2 and three breakfast group comparisons for each nutrient in each age group), 152 similar conclusions about breakfast group differences were reached with and without the added covariates (data not shown). For two nutrients (total fat in 12- to 17-year-olds and monounsaturated fat in 6- to 11-year-olds), one of the three breakfast group differences shifted from significant to not significant, and for two other nutrients (riboflavin and niacin in 12- to 17-year-olds) one of the three breakfast group differences shifted from not significant to significant.

To assess whether the differences in dietary intakes between the RTEC-breakfast and other-breakfast groups were associated with intake at breakfast *per se*, or whether they reflected differences in nutrient intake throughout the day, we compared the intakes of these two groups at the breakfast meal and during the rest of the day (Table 3). Breakfast intakes of energy, protein, niacin and folate did not differ between the groups. However, all the other nutrients differed. The RTEC-breakfast group had higher breakfast intakes of carbohydrate, sugars, fibre, vitamin D, thiamin, riboflavin, vitamin B_6_, Ca, P, Mg, Fe, Zn and K (all *P*< 0·0001), as well as vitamin B_12_ (*P*< 0·01). The other-breakfast consumers had higher intakes of total fat, saturated fat, monounsaturated fat, polyunsaturated fat, cholesterol and Na (all *P*< 0·001), as well as vitamins A and C (*P*< 0·01). During the rest of the day, intakes of energy, macronutrients and most micronutrients were similar between the groups, although riboflavin was slightly higher in the RTEC-breakfast group and Zn was slightly higher in the other-breakfast group (both *P*< 0·05).

**Table 3:** Nutrient intake of Canadian children and adolescents consuming breakfasts with and without ready-to-eat cereal (RTEC) at the breakfast meal and during the rest of the day† (Weighted means with their standard errors)

	Intake at breakfast	Intake during the rest of the day
	Other breakfast (*n* 6917)	RTEC breakfast (*n* 3890)	Other breakfast (*n* 6917)	RTEC breakfast (*n* 3890)
Nutrients	Mean	sem	Mean	sem	Mean	sem	Mean	sem
Energy (kJ)	1699	21	1761	25	7602	21	7540	25
Protein (g)	13·7	0·2	13·9	0·2	67·6	0·5	66·9	0·8
Fat (g)	14·1	0·3	8·4***	0·2	66·2	0·4	65·1	0·6
SFA (g)	5·1	0·1	3·7***	0·1	22·7	0·2	22·6	0·3
MUFA (g)	5·1	0·1	2·4***	0·1	26·4	0·2	25·6	0·3
PUFA (g)	2·3	0·05	1·1***	0·04	10·9	0·1	10·7	0·2
Cholesterol (mg)	72·0	2·4	23·3***	1·1	183·9	2·5	183·7	3·4
Carbohydrate (g)	57·6	0·8	74·3***	1·2	241·8	1·2	242·1	1·6
Sugars (g)	27·4	0·5	36·7***	0·7	108·2	1·0	108·0	1·5
Fibre (g)	2·5	0·05	3·6***	0·1	12·3	0·1	12·5	0·2
Vitamin A (μg RAE)	154·2	3·1	140·3**	3·1	494·8	8·2	502·9	10·2
Thiamin (mg)	0·37	0·01	0·87***	0·02	1·32	0·01	1·33	0·02
Riboflavin (mg)	0·52	0·01	0·65***	0·02	1·60	0·01	1·67*	0·03
Niacin (mg NE)	5·8	0·1	6·1	0·1	30·2	0·2	29·9	0·4
Vitamin B_6_ (mg)	0·29	0·01	0·50***	0·01	1·34	0·01	1·35	0·02
Folate (DFE)	93·9	1·5	92·8	2·0	372·8	3·7	368·3	4·4
Vitamin B_12_ (μg)	0·84	0·02	0·92**	0·02	3·2	0·1	3·2	0·1
Vitamin C (mg)	35·2	1·1	29·6**	1·5	120·4	2·1	117·6	2·6
Vitamin D (μg)	1·8	0·04	2·5***	0·05	4·2	0·1	4·4	0·1
Ca (mg)	258	5	277***	7	786	8	814	13
Fe (mg)	2·5	0·05	7·4***	0·2	10·6	0·1	10·7	0·1
Mg (mg)	59·5	0·9	78·6***	1·4	225·6	1·4	227·2	1·9
Zn (mg)	1·8	0·03	2·2***	0·04	9·1	0·1	8·8*	0·1
Na (mg)	624	15	553***	12	2658	24	2620	30
K (mg)	573	8	670***	11	2317	16	2318	23

RAE, retinol activity equivalents; NE, niacin equivalents; DFE, dietary folate equivalents.

Mean value was significantly different from that of the other-breakfast group: * *P*< 0·05, ** *P*< 0·01, *** *P*< 0·0001.

† Data were obtained from the Canadian Community Health Survey Cycle 2.2.

### Nutrient adequacy

Table 4 shows the prevalence of nutrient inadequacy from food sources alone and from the combination of food sources plus supplements (total intakes) for nutrients with an EAR. There was considerable variability across the nutrients in the prevalence of inadequacy, ranging from close to zero for niacin and riboflavin, to considerably higher proportions for Ca, Mg, vitamin A and vitamin D. Variability was also observed by breakfast group. For food and total intakes of vitamin D, Ca, Fe and Mg, the prevalence of inadequacy was significantly greater in breakfast non-consumers than in other-breakfast consumers, who in turn had a significantly greater prevalence of inadequacy than RTEC-breakfast consumers. For food and total intakes of vitamin A, P and Zn, and for total intakes of vitamin C, the prevalence of inadequacy was significantly greater among the breakfast non-consumers than among the groups of breakfast consumers, which did not differ. For food and total intakes of vitamin B_6_ and for food intakes of folate, the prevalence of inadequacy was significantly greater in breakfast non-consumers than in RTEC-breakfast consumers, neither of which differed from other-breakfast consumers. Also, for food and total intakes of thiamin, riboflavin, niacin and vitamin B_12_, there were no significant group differences in the prevalence of nutrient inadequacy.

**Table 4:** Prevalence of inadequate vitamin and mineral intakes (for nutrients with an estimated average requirement (EAR)) by breakfast group in Canadian children aged 4–18 years* (Mean values for percentage below the EAR with their standard errors)

	All breakfast group	Male breakfast group	Female breakfast group
	None (*n* 1471)	Other (*n* 6917)	RTEC (*n* 3890)	None (*n* 731)	Other (*n* 3335)	RTEC (*n* 2191)	None (*n* 740)	Other (*n* 3582)	RTEC (*n* 1699)
Nutrients	Mean	sem	Mean	sem	Mean	sem	Mean	sem	Mean	sem	Mean	sem	Mean	sem	Mean	sem	Mean	sem
Vitamin A																		
Food	50·0^a^	5·2	21·6^b^	2·4	17·8^b^	1·5	49·1^a^	7·4	18·7^b^	2·2	18·2^b^	2·8	51·1^a^	5·9	24·2^b^	3·4	17·3^b^	3·3
Food and supplements	45·4^a^	4·7	18·1^b^	1·9	14·0^b^	1·3	44·2^a^	6·5	15·7^b^	1·9	13·6^b^	2·0	46·0^a^	5·3	20·5^b^	2·6	14·3^b^	2·9
Thiamin																		
Food	6·1	3·9	0·5	0·3	0·1	0·1	8·2	5·6	0·1	0·1	0·1	0·1	4·4	5·5	1·0	0·5	0·1	0·1
Food and supplements	5·6	4·0	0·5	0·2	0·3	0·2	7·5	5·2	0·1	0·1	0·1	0·1	3·9	3·6	0·8	0·5	0·5	0·4
Riboflavin																		
Food	1·8	1·5	0·2	0·1	0·1	0·1	2·2	1·1	0·1	0·1	0·1	0·1	1·6	2·7	0·5	0·2	0·1	0·1
Food and supplements	1·7	1·2	0·3	0·1	0·3	0·2	2·2	0·9	0·1	0·1	0·1	0·1	1·4	2·3	0·4	0·2	0·5	0·4
Niacin																		
Food	0·0	0·0	0·0	0·0	0·03	0·04	0·0	0·0	0·0	0·0	0·02	0·06	0·0	0·0	0·0	0·0	0·04	0·09
Food and supplements	0·03	0·05	0·0	0·03	0·0	0·03	0·01	0·05	0·0	0·01	0·0	0·03	0·05	0·07	0·02	0·02	0·4	0·5
Vitamin B_6_																		
Food	10·7^a^	4·0	2·8^a,b^	0·9	0·7^b^	0·4	3·1	2·2	0·9	0·4	0·4	0·4	19·3^a^	6·8	4·5^a,b^	1·7	1·1^b^	0·6
Food and supplements	9·5^a^	3·3	2·4^a,b^	0·8	0·8^b^	0·4	2·6	2·3	0·8	0·4	0·4	0·4	16·6^a^	6·3	4·0^a,b^	1·4	1·4^b^	0·8
Folate																		
Food	23·8^a^	8·4	3·5^a,b^	1·2	2·7^b^	1·4	21·0	13·0	1·1	0·6	2·4	1·7	27·9	11·8	5·7	2·0	3·3	1·9
Food and supplements	21·3	8·2	3·1	1·0	2·5	1·2	18·5	10·4	1·0	0·5	1·8	1·3	24·3	11·2	5·1	1·6	3·3	1·8
Vitamin B_12_																		
Food	7·1	5·9	2·2	1·1	1·5	0·5	0·03	1·5	1·0	0·7	0·3	0·3	14·2	12·0	3·4	1·7	2·9	1·0
Food and supplements	6·2	5·2	2·0	0·9	1·4	0·5	0·03	0·8	0·9	0·5	0·2	0·2	12·6	10·9	3·0	1·5	2·7	0·9
Vitamin C																		
Food	11·8	4·5	3·3	0·6	3·1	1·0	4·5	3·9	4·1	0·9	4·5	1·7	19·0^a^	5·8	2·7^b^	0·8	1·3^b^	0·7
Food and supplements	10·6^a^	2·5	3·0^b^	0·4	2·6^b^	0·8	5·0	3·7	3·6	0·7	3·3	1·2	16·4^a^	5·1	2·2^b^	0·5	1·5^b^	0·7
Vitamin D																		
Food	95·7^a^	2·4	88·5^b^	1·3	81·6^c^	1·9	93·5^a^	4·8	81·1^a,b^	2·1	75·9^b^	4·4	98·6	2·4	95·3	1·3	88·7	3·6
Food and supplements	85·2^a^	2·7	70·3^b^	1·3	61·9^c^	1·9	82·6^a^	4·3	63·7^b^	1·8	56·8^b^	3·6	87·9^a^	2·3	76·6^b^	1·6	68·6^c^	2·9
Ca																		
Food	79·8^a^	3·7	55·1^b^	2·5	33·2^c^	3·0	76·7^a^	6·4	42·6^b^	3·4	25·8^c^	3·6	83·1^a^	4·3	66·9^b^	2·8	42·8^c^	5·2
Food and supplements	78·1^a^	3·9	51·9^b^	2·5	30·7^c^	2·8	75·4^a^	5·7	40·0^b^	3·5	23·6^c^	3·4	81·2^a^	4·1	63·3^b^	2·7	40·0^c^	4·7
Fe																		
Food	9·7^a^	2·6	2·9^b^	0·5	0·3^c^	0·1	1·9^a,b^	1·4	0·7^a^	0·2	0·1^b^	0·05	18·1^a^	4·9	5·1^b^	0·8	0·5^c^	0·2
Food and supplements	9·1^a^	2·4	2·5^b^	0·4	0·2^c^	0·1	2·0^a,b^	1·5	0·6^a^	0·1	0·1^b^	0·05	16·9^a^	4·5	4·4^b^	0·7	0·4^c^	0·2
Mg																		
Food	62·4^a^	3·0	26·5^b^	1·7	16·3^c^	2·0	56·4^a^	4·3	20·4^b^	2·1	13·1^b^	2·5	68·6^a^	5·6	32·2^b^	2·2	20·5^c^	2·6
Food and supplements	61·2^a^	3·2	25·4^b^	1·6	15·7^c^	2·0	55·0^a^	4·1	19·7^b^	2·1	12·6^b^	2·4	67·4^a^	5·4	31·3^b^	2·2	19·8^c^	2·6
P																		
Food	31·8^a^	6·0	13·6^b^	1·9	8·2^b^	1·4	11·5	9·3	4·8	1·2	4·3	1·1	53·6^a^	7·5	21·6^b^	3·2	13·5^b^	2·8
Food and supplements	31·1^a^	5·1	13·1^b^	1·8	8·0^b^	1·2	11·2	8·0	4·7	1·2	3·8	1·0	52·1^a^	8·3	20·8^b^	3·1	13·2^b^	2·7
Zn																		
Food	23·0^a^	6·1	6·8^b^	1·9	5·0^b^	1·4	11·3	5·1	2·5	0·8	2·5	1·2	36·4^a^	10·0	10·8^b^	3·4	8·6^b^	2·2
Food and supplements	21·9^a^	5·4	6·4^b^	1·8	4·9^b^	1·2	11·4	5·6	2·4	0·8	2·4	1·1	34·3^a^	9·1	10·4^b^	3·2	8·3^b^	2·1

RTEC, ready-to-eat cereal included with breakfast.

^a,b,c^Mean values within a row with unlike superscript letters were significantly different (*P*< 0·05; Bonferroni-adjusted *P*< 0·0167).

* Data were obtained from the Canadian Community Health Survey, Cycle 2.2. The prevalence of inadequate intakes was assessed as the proportion with usual intakes below the EAR for all nutrients except Fe, for which the probability method was used. Usual intakes were determined using the National Cancer Institute method^(^ ^26^ ^)^.

The prevalence of nutrient inadequacy cannot be determined for nutrients that have an adequate intake (AI) instead of an EAR, but the proportion with intakes above the AI can be considered adequate^(^ ^13^ ^)^. Over 99 % of all the three breakfast groups had Na intakes above the AI, with no significant differences among the groups. In contrast, although significantly higher proportions of both groups of breakfast consumers met the AI for fibre compared with breakfast non-consumers, the proportions were < 2 % for all the three groups. A somewhat similar situation existed for K, with 0·8 (sem 0·6) % of breakfast non-consumers, 4·9 (sem 0·7) % of other-breakfast consumers and 7·0 (sem 2·4) % of RTEC-breakfast consumers meeting the AI. These data are based on intakes from food sources alone, as only trivial amounts of Na, fibre and K were consumed as supplements.

### Potential risk of excessive intakes

With the exception of Na and Zn, the prevalence of the potential risk of excessive intakes (above the UL) for all three breakfast groups was < 1 % from food sources alone and < 2·5 % from the combination of food sources plus supplements. There were no differences among the breakfast groups. For Na, the prevalence of intakes above the UL from food sources only was approximately 90 %, and did not differ by breakfast group. The proportions of Zn intakes above the UL from food sources alone were 0·7 (sem 0·7), 4·8 (sem 0·9) and 8·0 (sem 1·8) % for breakfast non-consumers, other-breakfast consumers and RTEC-breakfast consumers, respectively. From food sources plus supplements, the proportions of Zn intakes above the UL were 1·3 (sem 0·8), 5·7 (sem 0·9) and 8·8 (sem 1·8) %. In both cases, breakfast non-consumers had a significantly lower prevalence than the two groups of breakfast consumers, which did not differ.

## Discussion

Underconsumed nutrients of potential public health concern in Canadian children and/or adolescents include Ca, P, Mg, K, vitamin A, vitamin D and fibre^(^ ^31^ ^,^ ^32^ ^)^. For these shortfall nutrients, intakes in the present study increased in a stepwise manner among breakfast non-consumers, other-breakfast consumers and RTEC-breakfast consumers (Ca, P, Mg, K, vitamin D and fibre) or were higher in both groups of breakfast consumers than in breakfast non-consumers (vitamin A). These findings are consistent with those of previous studies in other countries^(^ ^5^ ^–^ ^11^ ^,^ ^33^ ^)^. Of greater relevance than higher intakes, however, is our finding of stepwise decreases in the prevalence of dietary inadequacy for Ca, Mg, Fe and vitamin D among breakfast non-consumers, other-breakfast consumers and RTEC-breakfast consumers. This was the case for intakes from food sources alone and from the combination of food sources plus supplements. Furthermore, compared with breakfast non-consumers, the two groups of breakfast consumers had a lower prevalence of inadequacy for vitamin A and P (food sources alone and food sources plus supplements) and vitamin C (food sources plus supplements). Thus, breakfast consumption, and to a greater extent, RTEC breakfast consumption, was associated with meaningful decreases in inadequacy for key nutrients. Moreover, although the prevalence of inadequacy cannot be estimated for fibre and K, higher proportions of breakfast consumers met the AI for these nutrients.

In addition to the aforementioned shortfall nutrients, breakfast consumers had significantly higher intakes of several other nutrients. However, unlike the improvements in nutrient adequacy for the shortfall nutrients, higher intakes of the other nutrients did not always translate into significantly improved nutrient adequacy. In some cases, this was due to large standard errors (e.g. folate inadequacy was more prevalent in breakfast non-consumers than in breakfast consumers, but the difference was not significant). In other cases, it was related to the fact that the requirement is a cut-off point: when intakes of almost all the participants in a group exceed the EAR (e.g. riboflavin and niacin in breakfast non-consumers), higher intakes do not reduce the prevalence of inadequacy. Conversely, if a relatively large proportion has intakes near the requirement, small increments can substantially change the proportion that do (or do not) meet their requirements. This was the case for Mg in the present study, where relatively modest increases in intake were associated with substantial reductions in inadequacy between breakfast non-consumers, other-breakfast consumers and RTEC-breakfast consumers. These outcomes illustrate the importance of assessing nutrient adequacy, rather than focusing primarily on differences in nutrient intakes among groups.

In contrast to the impact of breakfast consumption on nutrient adequacy, supplement use did not appear to substantially reduce the prevalence of nutrient inadequacy, as can be seen by the generally similar prevalences of inadequacy from food sources alone *v.* from food sources plus supplements (Table 4). The exception was vitamin D, where the prevalence of nutrient inadequacy was 10 to 20 percentage points lower when supplement use was considered. However, it should be noted that the EAR for vitamin D reflects the estimated requirement for dietary intake in the absence of sunlight exposure^(^ ^34^ ^)^. Although Canadian children have an apparently high prevalence of low vitamin D intakes (irrespective of supplement use), assessment of the vitamin D status of Canadian children and adolescents using serum 25-hydroxyvitamin D does not suggest widespread vitamin D deficiency^(^ ^35^ ^)^. This indicates that the contribution of sunlight exposure is probably substantial.

Zn was the only nutrient for which the prevalence of the potential risk of excessive intakes was higher in breakfast consumers than in non-consumers. Intakes above the UL occurred almost exclusively in children aged 4 to 8 years (data not shown). In both Canada and the USA, the mean Zn intakes of children aged 1 to 3 years exceed the UL, and considerable proportions of children aged 4 to 8 years exceed the UL^(^ ^29^ ^,^ ^36^ ^–^ ^38^ ^)^. Based on these high prevalences, the absence of any evidence of adverse effects and the limited data used to set the UL, it has been suggested that the UL for Zn for young children was set at too low a level^(^ ^39^ ^)^. Further research is needed to better define a UL for children, but in the meantime efforts to reduce Zn intakes do not appear to be warranted.

Intakes of fat, saturated fat and cholesterol in RTEC-breakfast consumers appeared to be more closely aligned with current recommendations than were intakes of breakfast non-consumers or other-breakfast consumers. However, RTEC-breakfast consumers also consumed approximately 10 g/d more total sugars (the CCHS 2.2 data file does not differentiate between naturally occurring and added sugars). The difference in sugar intake occurred at the breakfast meal, since intakes during the rest of the day were virtually identical between the RTEC-breakfast consumers and other-breakfast consumers. Since approximately 95 % of those who consume RTEC at breakfast consume it with milk^(^ ^40^ ^)^, and Ca intake at breakfast averaged 120 mg higher among the RTEC-breakfast consumers than among the other-breakfast consumers, about half the higher sugar intake would have come from milk (120 mg Ca is provided by approximately 100 ml of milk, which contains approximately 5 g of lactose). Accordingly, the remaining difference (approximately 5 g) was probably added sugars provided by RTEC and/or the addition of sugar to cereal at the table. Some debate exists regarding the adverse effects of added sugars on health^(^ ^41^ ^–^ ^44^ ^)^; nevertheless, dietary guidance in both Canada^(^ ^45^ ^)^ and the USA^(^ ^46^ ^)^ recommends that intakes be reduced. While the entire food supply, including RTEC, warrants examination, the highest priority for reduction would appear to be foods/beverages with large amounts of added sugars and without meaningful amounts of other nutrients.

The strengths of the present study include a large sample size, use of rigorous methodology to examine nutrient adequacy, and consideration of supplement use. Furthermore, our data indicate that food choices at breakfast *per se* make important contributions to overall nutrient intake and adequacy, as most differences in daily intakes between those consuming RTEC *v.* other types of breakfasts were due to the intake at the breakfast meal (rather than during the rest of the day). These differences were probably due to nutrients present (or added as fortificants) in RTEC and the milk that normally accompanies it.

We also acknowledge a number of limitations. First, breakfast groups were defined based on intake on the day of the first 24 h dietary recall. Whether or not those classified as breakfast non-consumers, RTEC-breakfast consumers and other-breakfast consumers followed these specific breakfast patterns routinely could not be ascertained. However, the fact that a number of demographic characteristics differed among the three breakfast groups suggests some stability in the classification. In this regard, it should also be noted that variability in breakfast patterns would attenuate the observed group differences, rather than exaggerate them. A second limitation is that the potential for residual confounding cannot be excluded. In this regard, physical activity, ‘screen time’ and smoking could not be included as covariates in the primary analysis of nutrient intake. However, the pattern of differences among the breakfast groups was almost identical when these covariates were added to separate analyses of those aged 6 to 11 and 12 to 17 years, suggesting that their omission from the primary analysis did not have a meaningful impact on the results. Third, we also note that we assessed only two types of breakfasts: those that included or did not include RTEC. Other breakfast patterns may also be associated with variability in nutrient intakes and adequacy, and this warrants additional research. Finally, food intake data were self-reported.

### Concluding remarks

The results of the present study support our hypothesis that breakfast consumption in Canadian children and adolescents would be associated with a lower prevalence of nutrient inadequacy, and would have little or no impact on the prevalence of the potential risk of adverse effects from excessive nutrient intakes. A key finding was that improved adequacy was seen for nutrients of potential public health concern, including Ca, Mg, P, vitamin A and vitamin D, as well as higher intakes of K and fibre. Breakfasts that included RTEC were associated with further improvements for several of these nutrients, while also adding approximately 5 g to added sugar intake. The prevalence of skipping breakfast increased substantially with age. Given the impact on nutrient intake and adequacy, efforts to encourage and maintain breakfast consumption are warranted.
